# Stability of the Neurotensin Receptor NTS1 Free in Detergent Solution and Immobilized to Affinity Resin

**DOI:** 10.1371/journal.pone.0012579

**Published:** 2010-09-07

**Authors:** Jim F. White, Reinhard Grisshammer

**Affiliations:** National Institute of Neurological Disorders and Stroke, National Institutes of Health, Department of Health and Human Services, Rockville, Maryland, United States of America; Massachusetts Institute of Technology, United States of America

## Abstract

**Background:**

Purification of recombinant membrane receptors is commonly achieved by use of an affinity tag followed by an additional chromatography step if required. This second step may exploit specific receptor properties such as ligand binding. However, the effects of multiple purification steps on protein yield and integrity are often poorly documented. We have previously reported a robust two-step purification procedure for the recombinant rat neurotensin receptor NTS1 to give milligram quantities of functional receptor protein. First, histidine-tagged receptors are enriched by immobilized metal affinity chromatography using Ni-NTA resin. Second, remaining contaminants in the Ni-NTA column eluate are removed by use of a subsequent neurotensin column yielding pure NTS1. Whilst the neurotensin column eluate contained functional receptor protein, we observed in the neurotensin column flow-through misfolded NTS1.

**Methods and Findings:**

To investigate the origin of the misfolded receptors, we estimated the amount of functional and misfolded NTS1 at each purification step by radio-ligand binding, densitometry of Coomassie stained SDS-gels, and protein content determination. First, we observed that correctly folded NTS1 suffers damage by exposure to detergent and various buffer compositions as seen by the loss of [^3^H]neurotensin binding over time. Second, exposure to the neurotensin affinity resin generated additional misfolded receptor protein.

**Conclusion:**

Our data point towards two ways by which misfolded NTS1 may be generated: Damage by exposure to buffer components and by close contact of the receptor to the neurotensin affinity resin. Because NTS1 in detergent solution is stabilized by neurotensin, we speculate that the occurrence of aggregated receptor after contact with the neurotensin resin is the consequence of perturbations in the detergent belt surrounding the NTS1 transmembrane core. Both effects reduce the yield of functional receptor protein.

## Introduction

Structural and functional work on G-protein-coupled receptors [1], [2] such as the rat neurotensin receptor NTS1 [3] requires a reliable source of high-quality material. NTS1 occurs naturally at low levels; recombinant expression [4] and efficient purification methods are therefore needed to obtain pure NTS1. We established a bacterial expression system for the production of functional, membrane-inserted NTS1 [5] whereby the *Escherichia coli* maltose-binding protein (MBP) is fused to the receptor N-terminus. The *E. coli* thioredoxin at the receptor C-terminus was found to improve expression levels [6] and a C-terminal deca-histidine tag allowed the efficient capture of the NTS1 fusion protein by immobilized metal affinity chromatography (IMAC) [7]. We developed an automated procedure for the purification of fully functional NTS1 in detergent solution at the 3-milligram or 10-milligram level [8]. IMAC is the first purification step to enrich the NTS1 fusion protein. The second step makes use of a neurotensin (NT) column to remove contaminants remaining from the first purification step [8]. Receptors eluted from the NT column are pure and fully functional as assessed by radio-ligand binding and G-protein coupling experiments [8], [9].

As noted above, the contaminants present in the Ni-NTA column eluate can be removed by the subsequent NT column step. However, we also observed a considerable amount of NTS1 in the NT column flow-through. These receptors are initially detergent-soluble, do not bind ligand [8], and aggregate over time. Because the misfolded NTS1 species is unsuitable for biochemical work but constitutes a substantial fraction of the total amount of NTS1, we investigated the origin of the misfolded receptors to potentially take measures against NTS1 aggregation and to maximize the yield of functional NTS1. We detected some misfolded NTS1 in the Ni-NTA column eluate which could have arisen during biosynthesis in *E. coli* cells or by damage of intact receptors after solubilization with detergents. However, exposure of functional NTS1 in the Ni-NTA column eluate to the NT affinity resin generated additional misfolded receptor protein. Because NT stabilizes NTS1 in detergent solution, aggregation of NTS1 is likely caused by close contact of the receptor with the NT column matrix, thus reducing the overall yield of purified functional NTS1.

## Materials and Methods

### Materials

The tritiated agonist [^3^H]neurotensin ([^3^H]NT: [3,11-tyrosyl-3,5-^3^H(N)]-pyroGlu-Leu-Tyr-Glu-Asn-Lys-Pro-Arg-Arg-Pro-Tyr-Ile-Leu) was purchased from Perkin Elmer. Unlabeled neurotensin (NT) was purchased from Sigma. The detergents n-dodecyl-β-D-maltopyranoside (DDM) and 3-[(3-cholamidopyropyl)dimethylammonio]-1-propanesulfonate (CHAPS), and cholesteryl hemisuccinate Tris salt (CHS) were obtained from Anatrace.

### Expression of NTS1 in *Escherichia coli*

The NTS1 fusion protein MBP-T43NTR-TrxA-H10 (NTS1-624) consists of the *E. coli* maltose-binding protein (MBP, Lys1 to Thr366), followed by Gly-Ser, the N-terminally truncated rat neurotensin type I receptor NTS1 (T43NTR, Thr43 to Tyr424), three Ala residues, the *E. coli* thioredoxin (TrxA, Ser2 to Ala109), Gly-Thr and a decahistidine tag (H10). We refer here to NTS1-624 as NTS1. NTS1 was produced in *E. coli* as described [7], [8].

### Batch purification of NTS1 by immobilized metal affinity chromatography

All steps were carried out at 4°C or on ice. Solubilization of NTS1 was done as previously described (for details see reference [8]). Briefly, 10 gram of *E. coli* cells were processed in a final volume of 50 ml (final buffer composition: 50 mM Tris-HCl pH 7.4, 30% (v/v) glycerol, 200 mM NaCl, 1% (w/v) DDM, 0.6% (w/v) CHAPS, 0.12% (w/v) CHS, protease inhibitors: 70 µg/ml phenylmethylsulfonyl-fluoride (PMSF), 1 µg/ml leupeptin, 1.4 µg/ml pepstatin A, 5 µg/ml DNaseI and 5 mM MgCl_2_). After sonication (8 sec/gram of cells), unbroken cells and debris was removed by ultracentrifugation. Imidazole was then added to the supernatant (SN) to a final concentration of 50 mM. Binding of receptors to Ni-NTA Superflow resin (Qiagen, 5 ml settled resin) was done overnight in batch. The Ni-NTA column flow-through (NiFT) was collected for analysis. The Ni-NTA resin was washed four times with 20 ml of buffer NiA (50 mM Tris-HCl pH 7.4, 30% glycerol, 200 mM NaCl, 50 mM imidazole, 0.1% DDM, 0.5% CHAPS, 0.1% CHS). Elution was done with buffer NiB (NiA buffer with 200 mM imidazole).

### Purification of NTS1 by neurotensin affinity chromatography

The second purification step of the NTS1 fusion protein was performed using an Äkta Purifier system (GE Healthcare). The Ni-NTA column eluate (NiE) was diluted 2.85-fold with buffer NT0 (50 mM Tris-HCl pH 7.4, 30% glycerol, 0.1% DDM, 0.5% CHAPS, 0.1% CHS) to reduce the NaCl and imidazole concentrations to 70 mM and hence allow binding of NTS1 to the neurotensin column (NT column) [10]. The diluted NiE was loaded onto a NT column (4 ml resin packed into a XK16/60 column) [6] at a flow rate of 0.4 ml/min. After loading was completed, the NT column was washed with NT70 buffer (NT0 buffer with 70 mM NaCl; 20 column volumes). NTS1 was eluted from the NT column with buffer NT1K (NT0 buffer with 1 M NaCl).

### Radio-ligand binding assays

Agonist binding to the detergent-solubilized receptor fusion protein was analyzed with [^3^H]neurotensin ([^3^H]NT) (PerkinElmer) in assay buffer (50 mM Tris-HCl pH 7.4, 1 mM EDTA, 0.1% BSA, 0.004% bacitracin) containing detergent (0.1% DDM, 0.2% CHAPS, 0.04% CHS) for 1 hour on ice. The reaction volume was 150 µl. Non-specific [^3^H]NT binding was determined in the presence of 10 µM unlabeled NT. Separation of the receptor-ligand complex from free ligand (100 µl) was achieved by centrifugation-assisted gel filtration using Bio-Spin 30 Tris columns (Bio-Rad) according to the manufacturer's instructions. Samples were analyzed by liquid scintillation counting [8].

The apparent affinity of NT for solubilized NTS1 is reduced in the presence of Na^+^-ions [10], [11] and imidazole [10]. Therefore, NTS1 was diluted with dilution buffer (50 mM Tris-HCl pH 7.4, 1 mM EDTA, 0.1% DDM, 0.2% CHAPS, 0.04% CHS) prior to binding assays to minimize the carry-over of detergents and buffer components from the respective purification steps, and hence to lessen their effects on [^3^H]NT binding.

For saturation binding analyses, the [^3^H]NT concentration was varied from 0.3–10 nM. Data were analyzed by nonlinear regression using Prism software version 4 (GraphPad). A one-site binding equation was used to determine the values for maximal binding (B_max_) and the equilibrium dissociation constants (K_d_) with n denoting the number of independent experiments (SN: K_d_ = 1.09 nM±0.05, n = 6; NiE: K_d_ = 0.41 nM±0.03, n = 6; NTE: K_d_ = 0.46 nM±0.05, n = 4). Single-point ligand binding experiments were conducted at a [^3^H]NT concentration of 2 nM, and the amount of specifically bound [^3^H]NT was then corrected for fractional occupancy using the respective K_d_ value [12].

### Stability of NTS1 during purification

The stability of NTS1 in the SN, NiE and diluted NiE fractions at 4°C was assessed by measuring [^3^H]NT binding to receptors over time. Prior to each ligand binding assay, NTS1 was diluted into receptor dilution buffer. Agonist binding measurements were conducted at a [^3^H]NT concentration of 2 nM. Half-lives were calculated by nonlinear regression using the one phase exponential decay function (Prism software) (Table 1).

**Table 1:** Stability of NTS1 in the absence of agonist.

Sample	t½ (hrs) ± SE	% left after xx hrs
SN (n = 4)	124±13	91%±3, 24 hrs
NiE (n = 7)	138±8	97%±1, 6 hrs
diluted NiE (n = 6)	45±3	91%±1, 6 hrs

[^3^H]NT binding to the NTS1 fusion protein was recorded over time and half-lives were calculated. The amount of functional NTS1 remaining after 6 hrs (NiE and diluted NiE) and after 24 hrs (SN) was estimated from one phase exponential decay fits. Abbreviations: SN, supernatant after ultracentrifugation; NiE, Ni-NTA column eluate.

To investigate the effect of NT on the stability of NTS1 in the diluted NiE fraction, we used a modified stability test. Receptors were diluted to a concentration of 9 nM into NT70 buffer containing [^3^H]NT at a final concentration of 10 nM. At various time points, 100 µl of the reaction mixture was processed over Bio-Spin 30 Tris columns. Non-specific binding was determined in the presence of 10 µM unlabeled NT. For comparison, we assessed the stability of NTS1 in diluted NiE in the absence of NT. For this, NTS1 was diluted into NT70 buffer to 10 nM. At the required time points, [^3^H]NT was added to a final concentration of 10 nM (reducing the receptor concentration to 9 nM). After one hour incubation on ice, 100 µl of the reaction mixture was processed over Bio-Spin 30 Tris columns.

### Amido Black analysis

The protein content was measured according to the method of Schaffner and Weissmann [13] with bovine serum albumin as the standard.

### SDS-PAGE and Western blot analysis of *E. coli* cells and SN samples

*E. coli* cells were collected by centrifugation. The cell pellet was suspended in H_2_O and the volume was immediately determined. SDS was then added to a final concentration of 2% followed by incubation for 1 hour at ambient temperature. 4× LDS reducing sample buffer (Invitrogen) was then added to give 1× sample buffer concentrations. The final volume was ∼20% of the original cell culture volume. After centrifugation at 16,000×g for 30 min, the supernatant was used for gel electrophoresis.

The protein supernatant samples after detergent solubilization and ultracentrifugation (SN samples, see section “Batch purification of NTS1 by immobilized metal affinity chromatography”) were adjusted with 4× LDS reducing sample buffer to give 1× sample buffer concentrations and further diluted if required.

Proteins were separated by SDS-PAGE on NuPAGE 4–12% Bis-Tris gels in MES buffer and transferred to ImmobilonP polyvinylidene fluoride membranes (Millipore) in transfer buffer (25 mM Tris base pH 8.3, 150 mM glycine, 0.05% SDS, 5% methanol) using the Hoefer semi-dry blotter (100 mA, 40 min). After blocking in phosphate buffered saline (PBS) supplemented with 0.05% Tween-20 and 3% BSA, the ImmobilonP membranes were incubated with INDIA HisProbe-HRP (1∶5000, Pierce) for 45 min. The membranes were then washed twice for 5 min with PBS, 0.05% Tween-20, and once for 5 min with PBS. The subsequent incubation with SuperSignal West Pico chemiluminescent substrate (Pierce) was done according to the manufacturer's instructions. Membranes were exposed to x-ray film (Kodak Biomax XAR) for defined time intervals.

For ImageJ quantification by Western blot of NTS1 in *E. coli* cells and the SN samples, 10–90 ng of purified reference NTS1 fusion protein was loaded per lane.

### SDS-PAGE of NiE, NTFT and NTE

The NiE, NTFT and NTE samples were precipitated prior to gel electrophoresis by addition of methanol and chloroform, and air dried. Proteins were then dissolved for 1 hour in a 2% SDS solution at 0.3–1.3 mg/ml. Finally, 4× LDS reducing sample buffer (Invitrogen) was added to give 1× sample buffer concentrations. Proteins were separated on NuPAGE 4–12% Bis-Tris gels in MES buffer and stained with SimplyBlue SafeStain (Invitrogen). The Perfect Protein Marker (15–150 kDa) used as standard was purchased from Novagen.

For ImageJ quantification of NTS1 in the NiE, NTFT and NTE fractions, a range of purified reference NTS1 fusion protein (0.5–4 µg per lane) was included on Coomassie-stained gels.

### ImageJ analysis of films and Coomassie-stained gels

Developed films and Coomassie-stained gels were scanned using a HP ScanJet 8300 scanner with the resolution set to 600 pixels/inch. The scans were saved as tif files and imported into the ImageJ software package (Rasband, ImageJ, U.S. National Institutes of Health, Bethesda, Maryland, USA, http://rsb.info.nih.gov/ij/, 1997–2008) [14]. The intensities of the protein bands were plotted and integrated to give area values.

The ng/µg-amounts of NTS1 in cell samples, SN, NiE, NTFT and NTE were then calculated as follows.

NTS1 in *E. coli* cells and SN (Western blot, film): A standard curve was constructed from purified reference NTS1 fusion protein by relating the ImageJ-derived areas of the reference protein to the known ng-amounts loaded per lane. The ng-amount of the target NTS1 was calculated by comparing the ImageJ-derived area to the standard curve by linear regression.

NTS1 in the NiE, NTFT and NTE fractions: A standard curve was constructed from purified reference NTS1 fusion protein by relating the ImageJ-derived areas of the reference protein to the known µg-amounts loaded per lane. The µg-amount of the target NTS1 was calculated by comparing the ImageJ-derived area to the standard curve by linear regression. This approach assumes that Amido Black binds equally well to bovine serum albumin, used as standard for determination of the protein content, and to the purified reference NTS1 fusion protein. Coomassie Blue is expected to bind equally well to the purified reference NTS1 fusion protein and to the target NTS1. The comparison of the ImageJ-calculated µg-amounts of NTS1 with amounts determined by [^3^H]NT saturation binding experiments allowed to estimate the percentage of functional NTS1 in the NiE, NTFT and NTE.

### Data compilation in Table 2

**Table 2:** Functional and misfolded NTS1 during purification.

		By Ligand binding	By Image J analysis of gels	By Specific binding	Average across row using independent parameters	Percentage relative to protein species in NiE (%)
		A	B	C	D	
	NiE		80%^4^ fct.	36%^3^		
1	Functional NTS1	0.323^5^←0.355^1^	0.323^5^←0.355^1^^,^ 6	0.301^5^←0.331^3^	0.312	100
2	Misfolded NTS1	0.032^5^	0.032^5^+0.088^6^	0.030^5^	0.119	100
3	Total NTS1	NA	0.443^2^	NA	0.443	100
4	Contaminants	NA	0.477^7^	NA	0.477	100
5	Total protein	NA	0.920^8^	0.920^8^	0.920	100
	NTFT		4%^4^ fct			
1	Functional NTS1	0.007^1^	0.007^1^	NA	0.007	2
2	Misfolded NTS1	NA	0.164^6^	NA	0.164	138
3	Total NTS1	NA	0.171^2^	NA	0.171	39
4	Contaminants	NA	0.359^7^	NA	0.359	75
5	Total protein	NA	0.530^8^	0.530^8^	0.530	58
	NTE		101%^4^ fct	96%^3^		
1	Functional NTS1	0.124^1^	0.124^1^	0.124^3^	0.124	40
2	Misfolded NTS1	NA	NA	0.006	0.006	5
3	Total NTS1	0.124	0.123^2^	0.130^8^	0.126	28
4	Contaminants	NA	0.007^7^	NA	0.007	2
5	Total Protein	0.124	0.130^8^	0.130^8^	0.127	14

Three different ways (columns A–C) were used to estimate the amounts of functional NTS1, misfolded NTS1 and contaminants in the NiE, NTFT and NTE fractions. All values in columns A–D are given in mg quantities. Abbreviations: NiE, Ni-NTA column eluate; NTFT, flow-through of NT column; NTE, NT column eluate; NA, not applicable.

1 The amount of functional NTS1 in the NiE, NTFT and NTE fractions was determined by [^3^H]NT binding (see Tables 3 and 4). For example, the NiE contained 3678 pmoles or 0.355 mg functional NTS1.

2 The total amount of NTS1 protein in the NiE, NTFT and NTE fractions was determined by ImageJ analysis of Coomassie-stained gels (Table 4).

3 The amount of functional NTS1 in the NiE and NTE fractions was derived from the respective specific [^3^H]NT binding values (in pmol/mg, Table 3) compared to the theoretical value of 10363 pmol/mg. For example, the NiE fraction has a specific binding value of 3724 pmol/mg and hence 36% (3724/10363) of the total protein in NiE (0.92 mg) is functional NTS1 (0.331 mg).

4 The amount of functional NTS1 was estimated from data in Table 4.

5 The diluted NiE is 91% functional after 6 hrs (Table 1). This decrease of the amount of functional NTS1 is indicated by a left pointing arrow. The corresponding amount of misfolded NTS1 is listed.

6 The amount of functional and misfolded NTS1 was calculated from the total NTS1 amount in the respective fractions considering the proportion of functional receptors (Table 4). For example, the NiE contained 0.443 mg total NTS1 which is 80% functional^4^ i.e. 0.355 mg functional NTS1 and 0.088 mg misfolded NTS1.

7 The amount of contaminants was calculated by subtracting the total NTS1 amount from the total protein content of a given fraction (Table 4).

8 The total protein content of the NiE, NTFT and NTE fractions was determined by the Amido Black method.

The columns and rows of Table 2 are numbered and a particular cell within Table 2 is referred to as [column/row]. Column A lists the amount of functional NTS1 in the NiE, NTFT and NTE fractions determined by [^3^H]NT binding experiments (see Tables 3 and 4) and stability measurements (Table 1). For NiE, the amount of functional NTS1 was corrected for the loss of functional receptors during the 6 hour time interval needed for completion of the NT column step (Table 1). This decrease of the amount of functional NTS1 in the NiE fraction is indicated by a left pointing arrow in [A/1]. The corresponding amount of misfolded NTS1 is listed in [A/2]. The analysis of [^3^H]NT binding to NTS1 in the NTFT and NTE fractions was done 12 hours after these fractions became available. No decrease of ligand binding was observed for NTS1 in the NTE over a time period of 12 hours. Column B lists information from ImageJ analysis of Coomassie-stained gels (Table 4), [^3^H]NT binding assays (Tables 3 and 4) and protein content determination by the Amido Black method (Table 3). [B/3] gives the total amount of NTS1 (Table 4). Subtracting from [B/3] the amount of functional NTS1 ([B/1], Tables 3 and 4) gives the amount of misfolded NTS1 [B/2]. For NiE, the amount of functional NTS1 [B/1] was corrected for the loss of functional receptors during the 6 hour time interval needed for completion of the NT column step (left pointing arrow in [B/1], see Table 1); the respective amount of misfolded NTS1 is listed in [B/2]. The amount of contaminating protein [B/4] was calculated by subtracting the total amount of NTS1 [B/3] from the entire protein content of the respective fraction determined by the Amido Black method [B/5]. Column C uses the specific [^3^H]NT binding values, calculated from [^3^H]NT binding and from the protein content by the Amido Black method (Table 3), to determine the amount of functional NTS1 in the NiE and NTE fractions. For this, the respective specific binding values (Table 3) are compared to the theoretical value of 10363 pmol/mg (see Table 3) to give the percentage functional NTS1 per total protein. The amount of functional NTS1 [C/1] was then calculated from the total protein content of the respective fraction (Table 3) and the percentage of functional NTS1. For NiE, the amount of functional NTS1 [C/1] was corrected for the loss of functional receptors during the 6 hour time interval needed for completion of the NT column step (left pointing arrow in [C/1], Table 1); the respective amount of misfolded NTS1 is listed in [C/2].

**Table 3:** Purification of NTS1.

	Volume (ml)	Functional receptor (pmoles)	Protein (mg)	Specific binding (pmol/mg)	Recovery (% by [^3^H]NT binding)	Recovery (% by Amido Black assay)
SN (n = 6)	44.6±0.2	5770±352	394±18	13.0±0.4	100	100
NiFT (n = 6)	50.2±0.2	761±60	ND	ND	13±1	ND
NiE (n = 4)	14.2±0.3	3926±178	1.00±0.10	3724±354	73±3	0.3±0.03
NiE loaded	13.4±0.4	3678±165	0.92±0.06		100	100
NTFT (n = 3)	41.8±0.9	70±4	0.53±0.03		2±0.1	59±2
NTE (n = 4)	3.5±0.0	1286±52	0.13±0.01	9911±826	35±0.5	14±0.5

Data are given ± SE. The volume of the NTFT is larger than that of the NiE because of the dilution applied to reduce the NaCl and imidazole concentrations. Recovery data of the NiFT and NiE relate to SN, whereas recovery data of the NTFT and NTE relate to the NiE loaded onto the NT-column. A theoretical value for specific binding of 10363 pmol/mg is calculated for NTS1 (molecular mass of 96.5 kDa) assuming one ligand binding site per receptor molecule. Abbreviations: SN, supernatant after ultracentrifugation; NiFT, flow-through of Ni-NTA column; NiE, Ni-NTA column eluate; NTFT, flow-through of NT column; NTE, NT column eluate; ND, not determined.

**Table 4:** Percentage of functional NTS1.

	Total protein by Amido Black (mg)	Total NTS1 protein (mg)	Functional NTS1 (mg)	Percent functional
NiE loaded	0.92±0.06	0.443±0.036 (6 gels)	0.355	80%
NTFT	0.53±0.03	0.171±0.007 (4 gels)	0.007	4%
NTE	0.13±0.01	0.123±0.009 (3 gels)	0.124	101%

The total protein content of the NiE, NTFT and NiE fractions was determined by the Amido Black method (Table 3). The amount of total NTS1 in the NiE, NTFT and NiE fractions was determined by ImageJ analysis of Coomassie-stained gels. The number of functional NTS1 in pmoles (Table 3) was converted into a milligram value using an NTS1 molecular mass of 96.5 kDa. The percent functionality was calculated as [(mg NTS1 by ligand binding analysis)/(mg NTS1 by SDS-PAGE analysis)]*100.

## Results

**Figure 1:**
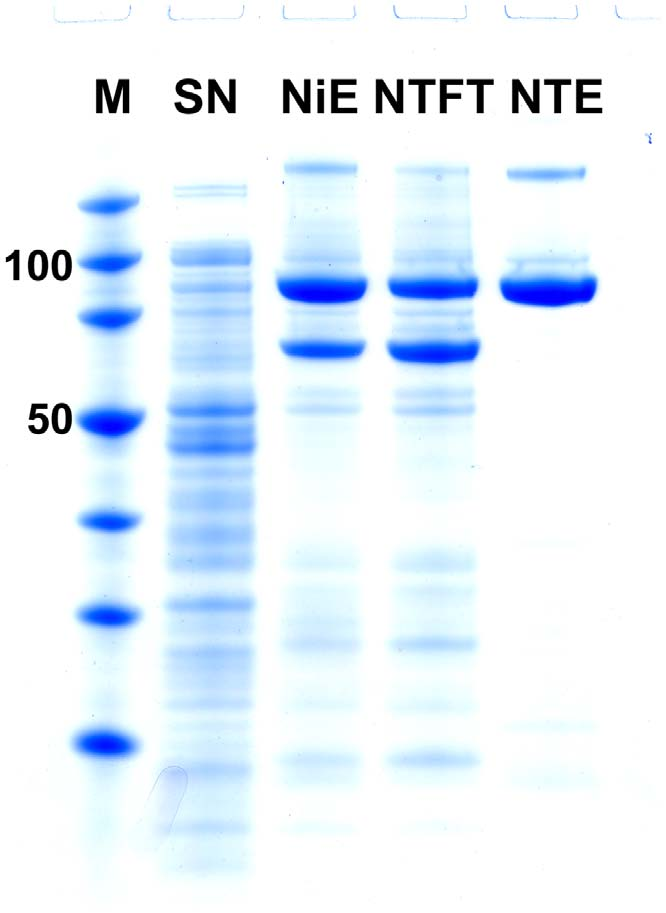
Purification of NTS1. The progress of purification was monitored by SDS-PAGE (NuPAGE 4–12% Bis-Tris gel, Invitrogen, 1× MES SDS buffer) and SimplyBlue staining. Lane M: Novagen Perfect Protein Marker (15–150 kDa); lanes SN, NiE and NTFT: 5 µg of protein per lane; lane NTE: 3 µg of protein. Abbreviations are as in Table 3.

The progress of purification of NTS1 by IMAC followed by a neurotensin (NT) column is shown in Figure 1 and in Table 3. The Ni-NTA column eluate (NiE) contained two major proteins, NTS1 and a 67 kDa protein identified as glucosamine-fructose-6-phosphate aminotransferase by mass spectrometry and N-terminal sequence analysis (data not shown). The subsequent NT column step efficiently removed the contaminants; however, the NT column flow-through (NTFT) also contained a considerable amount of NTS1 which no longer interacted with NT. NTS1 in the NTFT severely aggregated over time (data not shown) indicating that this receptor species was misfolded. Because correctly folded receptors bind radio-ligand, but misfolded receptors do not, one can approximate at the various purification steps the percentage of functional receptors by comparing [^3^H]NT binding data with estimates of the total amount of NTS1 (functional and misfolded). This approach allowed identifying the source of misfolded receptors found in the NTFT.

If incorrectly folded receptors arise in *E. coli* during NTS1 biosynthesis, detergent extraction would result in a mixture of misfolded and correctly folded receptor protein in the supernatant fraction (SN) after solubilization and ultracentrifugation. Because heavily aggregated receptors would not be amenable to solubilization with mild detergents such as DDM and CHAPS used in this study, the presence of detergent-solubilized misfolded NTS1 would indicate only minor receptor defects compromising agonist binding. To investigate this hypothesis, we performed [^3^H]NT saturation binding analyses to determine the amount of functional NTS1 in *E. coli* cells and in the SN fraction. We then compared these data to those obtained from Western blots and ImageJ analyses to estimate the total amount of NTS1 protein in *E. coli* cells and in the SN using purified NTS1 fusion protein for the standard curve. We found that the amount of NTS1 determined by [^3^H]NT saturation binding (i.e. functional receptors) was greater (>130%, data not shown) than that determined by Western blot analysis (total NTS1 protein), suggesting that NTS1 is “more than 100% functional” in *E. coli* cells and in the SN. One explanation for this observation would be incomplete transfer of NTS1 from the SDS-gels onto polyvinylidene fluoride membranes resulting in an underestimate of the total receptor protein. Indeed, we observed by silver staining residual NTS1 protein in gels after the Western blot procedure using purified NTS1 protein for test purposes (data not shown). Although we cannot conclude whether some misfolded NTS1 protein originated during expression in *E. coli* cells, and if so to what extent, our data do not indicate the presence of large amounts of misfolded receptors in *E. coli* cells or SN.

Because the NiE, NTFT and NTE fractions were enriched in NTS1 and contained only a limited number of contaminants (Figure 1), receptors were directly visualized on SDS-gels after Coomassie-staining eliminating the need of Western blotting for NTS1 identification. The quantification of total NTS1 protein was achieved by ImageJ analysis of SDS-gels comparing the intensities of Coomassie-stained receptors with those of defined amounts of purified NTS1 standards (Table 4). The amount of functional receptors was estimated by [^3^H]NT saturation binding (Tables 3 and 4). We found that 80% of NTS1 in the NiE was functional. The NTFT contained misfolded receptor, and the NTE consisted of pure fully functional NTS1 (Table 4).

Misfolded NTS1 may arise during purification when correctly folded receptors suffer damage by exposure to detergents and/or to potentially destabilizing buffer compositions; these effects would result in an increase of misfolded NTS1 over time. To account for this possibility, we determined the stability of NTS1 in SN, NiE and diluted NiE (Table 1). Receptors in these fractions are exposed to different concentrations of detergent, lipid and salt in the absence of NT, and we observed a decrease in the amount of functional NTS1 over time. The stability of NTS1 was comparable in the SN and NiE fractions indicating that changes in detergent concentration (DDM 1%→0.1%; the CHAPS and CHS concentrations change much less) and possibly in lipid content had no profound effect on the receptor half-lives at these purification stages. However, lowering the NaCl and imidazole concentrations from 200 mM (NiE) to 70 mM (diluted NiE) reduced the half-life of NTS1 by about 3-fold. The concomitant change in receptor concentration was 2.85-fold (see Materials and Methods) and hence is less likely to explain the reduced NTS1 stability in the diluted NiE fraction compared to NiE. The solubilization and IMAC step required 24 hours for completion, while the NT column step was completed after 6 hours. During these time intervals, less than 10% of receptor functionality was lost at each step (Table 1). Although small, we accounted in Table 2 for the amount of misfolded NTS1 generated by exposure to detergent and various buffer compositions.

Table 2 shows the estimates of functional NTS1, misfolded NTS1, contaminants and total protein in the NiE, NTFT and NTE fractions. We used three different ways (see columns A–C in Table 2) to describe those parameters; we realize some interdependence because of overlapping use of data from radio-ligand binding assays and from Amido Black protein determination. Column A shows the amounts of functional NTS1; for NiE, this was corrected for receptor instability in buffer with low NaCl content over the time period needed for completion of the NT column step (Table 1). Column B lists information from ImageJ analyses of SDS-gels which gives an estimate of the total amount of NTS1 by comparing the Coomassie Blue intensities of NTS1 bands in the respective purification fractions to a standard curve with purified receptor. By considering radio-ligand binding data, the amount of misfolded NTS1 was calculated in each fraction. The amount of contaminants was determined as the difference between the total protein content of a purification fraction and the respective total NTS1 protein. Column C uses the specific [^3^H]NT binding values of the NiE and NTE fractions, calculated from [^3^H]NT binding and from the protein content by the Amido Black method (Table 3), to determine the amount of functional NTS1.

**Figure 2:**
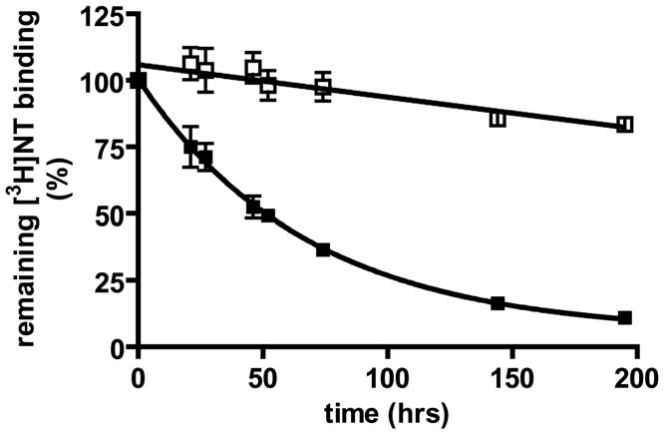
Stability of NTS1 in NT70 buffer with and without agonist. Receptors were incubated with [^3^H]NT (open squares) or kept in NT70 buffer without agonist (filled squares) at 4°C. [^3^H]NT binding was determined at the indicated time points. The data shown are from 2 independent experiments.

The average estimates of functional NTS1, misfolded NTS1, contaminants and total protein in the NiE, NTFT and NTE fractions are listed in column D of Table 2. In the NiE fraction, ∼25% of the total NTS1 protein or 0.12 mg was misfolded. In contrast, the NTFT and NTE fractions contained 0.17 mg of misfolded NTS1, an increase to 143% (Tables 2 and 5). This was surprising because on average we could not account for ∼30% of the total protein, contaminants and NTS1 protein in the NTFT and NTE fractions compared to the NiE load (Table 5). Hence this increase of misfolded NTS1 rather than the expected decrease by ∼30% indicated that misfolded NTS1 was generated during the NT column step. To investigate whether NTS1 assumed an aggregation-prone conformation by virtue of binding its agonist, we performed stability measurements of NTS1 in buffer NT70 in the presence or absence of NT. NTS1 was considerably more stable with ligand bound (Figure 2) suggesting that the event of agonist binding *per se* on the NT column was unlikely to contribute to the amount of misfolded NTS1 found in the NTFT. Instead, the close proximity of the receptor to the NT column resin may lead to receptor destabilization.

**Table 5:** Summary of NTS1 purification.

	Protein (mg)	Protein (percent)	Unaccounted (mg)	Unaccounted (percent)
**NiE**				
Functional NTS1	0.312	100		
Misfolded NTS1	0.119	100		
Total NTS1	0.443	100		
Contaminants	0.477	100		
Total protein	0.920	100		
**NTFT+NTE**				
Functional NTS1	0.131	42	−0.181	58
Misfolded NTS1	0.170	143	+0.051	(43)
Total NTS1	0.297	67	−0.146	33
Contaminants	0.366	77	−0.111	23
Total protein	0.657	71	−0.263	29

The percent values refer to the respective protein species in the NiE.

## Discussion

Previously, we reported a robust two-step purification procedure for NTS1 using IMAC followed by a NT column step [8]. Whilst the NT column eluate contained functional receptor protein, the NT column flow-through contained misfolded NTS1. Our data presented here point towards two ways by which misfolded NTS1 may be generated.

First, correctly folded NTS1 suffers damage by exposure to detergent and by changes in buffer composition as seen by the loss of [^3^H]NT binding over time in the SN, NiE and diluted NiE fractions (Figure 2, Table 1). For the Ni-NTA column step, 86% of [^3^H]NT binding sites can be found in the combined NiFT (13%) and NiE (73%) fractions when compared to the SN (Table 3). Because the amount of functional NTS1 in the SN fraction is reduced by 9% over the 24 hour time period needed for completion of solubilization and the IMAC step (Table 1), almost all [^3^H]NT binding sites are accounted for, suggesting that immobilization *per se* of NTS1 via the C-terminal histidine tag does not result in substantial receptor destabilization.

Second, the NT column step generates additional receptor protein which is misfolded, thereby reducing the yield of pure, functional NTS1. The use of a NT column is required to efficiently remove contaminants contained in the eluate of the Ni-NTA column. The following four points may contribute to the instability of NTS1 during the NT column step:

- Binding of NT to NTS1 may cause an NTS1 conformation which is structurally unstable: Exposure of purified NTS1 to agonist allows the receptor to assume a conformation capable of catalyzing nucleotide exchange at Gαqβ_1_γ_2_ in detergent solution [9]. Currently it is unknown whether the conformation of agonist-loaded NTS1 in the absence of G-protein is the same or is different from the conformation of NTS1 in complex with NT and G-protein (see [15]), and hence we cannot comment on the biochemical properties of such specified NTS1 states. However, NT binding *per se* is unlikely to trigger misfolding of NTS1 on the NT column because agonist-loaded NTS1 is more stable than unoccupied NTS1 in NT70 buffer (Figure 2). Similar observations were made with NTS1 directly after solubilization [16] and with structurally unstable constitutively active receptor mutants that could be stabilized by agonist addition [17]–[19].
- Close proximity of NTS1 to the NT column matrix may destabilize NTS1: NT is attached to the NT column matrix via an N-terminal biotin group bound to immobilized tetrameric avidin [6]. The NT binding site of NTS1 has been mapped by mutagenesis experiments and modeling studies to residues in TM6, TM7 and the extracellular loop 3 [20]–[22]. We speculate that binding of NTS1 to the NT column therefore brings the receptor into close contact with avidin and the Sepharose matrix possibly causing perturbations in the detergent belt surrounding the NTS1 transmembrane core leading to subsequent denaturation. However, binding to Ni-NTA resin of the distant C-terminal histidine tag, which is separated from the NTS1 tramsmembrane core by the NTS1 C-terminus of ∼40 amino acid residues and the *E. coli* thioredoxin spacer does not cause appreciable receptor denaturation (see above).
- Aggregation of NTS1 on the NT column may result from a high local receptor concentration: The NT column has a theoretical ligand density of 100–120 µM [6]. If NTS1 binds to immobilized NT ligands as they become available during the loading process from the top to the bottom of the column, NTS1 would concentrate locally to ∼12 mg/ml (120 µM, molecular mass of 96.5 kDa for the NTS1 fusion protein). In contrast, the concentration of NTS1 bound to Ni-NTA resin would be lower because of the reduced apparent capacity of the IMAC column (<0.5 mg receptor fusion protein / ml resin or 5 µM; see references [8], [23]). The concentrations of NTS1 in the SN, NiE and NTE fractions range from 0.13–0.37 µM, respectively (Table 3), and the receptor stability test in the diluted NiE fraction (Figure 2) was done at an NTS1 concentration of only 9–10 nM. Hence, the use of the NT column constitutes a step in the presented purification scheme at which NTS1 may concentrate to a threshold initiating receptor denaturation. However, use of a NT column with a theoretical ligand density of 20 µM rather than 100–120 µM gave similar results (data not shown) suggesting that local high NTS1 concentrations were unlikely to cause receptor aggregation.
- The observed destabilization may reflect properties specific to NTS1: In contrast to NTS1, purification of an adenosine A2a receptor fusion protein by antagonist xanthine amine congener affinity chromatography with an estimated resin capacity of 14 mM did not lead to substantial amounts of A2a receptor protein in the antagonist column flow-through [23]. This may reflect the fact that the A2a receptor is inherently more stable than NTS1 [16], [24].

Several recombinant G-protein-coupled receptors have been purified for biochemical and structural work at large scale by affinity tag chromatography, followed in some cases by receptor-specific ligand columns. Examples are a Flag epitope at the receptor N-terminus for use with an M1 antibody column [25]–[27], or a poly-histidine tail at the receptor C-terminus for immobilized metal affinity chromatography [7], [8], [23], [25], [28]–[34]. A 1D4 antibody column [35], [36] recognizing the extreme C-terminus of bovine rhodopsin has been used for the single-step purification of rhodopsin [37], [38] and of a β-adrenergic receptor with the 1D4 epitope tag fused to its C-terminus [39]. Ligand-specific purification steps have been reported for the β_1_- and β_2_-adrenergic receptors (antagonist alprenolol column) [25]–[27], [32], [33], the A2a receptor (antagonist xanthine amine congener column) [23], a receptor for pituitary adenylate cyclase-activating polypeptide (biotinylated PACAP38) [40], and NTS1 (agonist neurotensin column) [8]. A destabilizing effect by the use of a receptor-specific ligand column has not been reported in literature; such an effect, as seen for NTS1, would certainly depend on a number of variables such as the intrinsic stability of the GPCR, the exact buffer and detergent conditions used during solubilization and purification, and measures taken to increase receptor stability (see [41]). Remarkably, a mutant β_2_-adrenergic receptor [28] and the A2a receptor [30] have been purified to homogeneity by the sole use of histidine tag chromatography steps; and the crystal structures have subsequently been determined. A similar purification approach is currently being evaluated for NTS1 to maximize receptor yields.
